# Intravenous lidocaine suppresses fentanyl-induced cough in Children

**DOI:** 10.1186/1745-9974-9-20

**Published:** 2013-08-15

**Authors:** Agreta Gecaj-Gashi, Zorica Nikolova-Todorova, Vlora Ismaili-Jaha, Musli Gashi

**Affiliations:** 1 Clinic of Anesthesiology & Intensive Care, University Clinical Centre of Kosova, 10000, Prishtina, Republic of Kosova; 2 Clinic of Anesthesiology & Reanimatology and Intensive Care, University Clinical Centre of Skopje, Skopje, Republic of Macedonia; 3 Pediatric Clinic, University Clinical Centre of Kosova, Prishtina, Republic of Kosova; 4 Emergency Center, University Clinical Centre of Kosova, Prishtina, Kosova

**Keywords:** Lidocaine, Cough, Fentanyl, Children, General anesthesia

## Abstract

**Objective:**

Fentanyl-induced cough is usually mild and transitory, but it can be undesirable in patients with increased intracranial pressure, open wounds of the eye, dissecting aortic aneurism, pneumothorax, and reactive airway disease. The aim of this study is to evaluate the efficacy of lidocaine in suppressing fentanyl-induced cough in children during induction in general anesthesia.

**Methods:**

One hundred and eighty-six children of both sexes, aged between 4–10 years, ASA physical status I and II, and scheduled for elective surgery, were recruited for the study. Patients with a history of bronchial asthma, obstructive pulmonary disease, or infections of the respiratory tract were excluded. Patients were randomly allocated to three equal groups (n = 62) to receive 1.0 mg/kg lidocaine (Group I), 0.5 mg/kg lidocaine (Group II), or placebo (equal volume of 0.9% saline; Group III). Each was administered over 5 s one minute before intravenous (IV) administration of fentanyl 2−3 μg/kg during induction in general anesthesia. The severity of coughing was graded by counting the number of episodes of cough: mild (1−2), moderate (3−4) or severe (5 or more).

**Results:**

Demographic information was comparable between groups. The most frequent coughing was observed in the placebo group (Group III; 43.5%), of whom 4.8% (three patients) were graded with severe cough. In Group II, 22.6% patients had cough, of which 1.6% (one patient) was graded as severe. In Group I, 16.1% patients had cough, none of whom were graded as severe.

**Conclusion:**

Our results demonstrate that IV lidocaine can markedly suppress fentanyl-induced cough in children, even in doses as low as 0.5 mg/kg.

## Introduction

Opioids are known for their antitussive effect, but often, intravenous administration of fentanyl during the induction of anesthesia paradoxically induces cough [1-5], although the exact mechanisms of fentanyl-induced cough still remain unclear. Fentanyl is commonly used as a preinduction adjunct in children. Fentanyl-induced cough is quite common and benign, but sometimes it may be explosive and can be associated with an increase in intraocular, intracranial, and intra-abdominal pressures, which may require immediate treatment [3,4,6]. Intravenous administration of lidocaine suppresses the cough reflex during endotracheal intubation, extubation, bronchography, bronchoscopy, and laryngoscopy [7-9]. Oshima et al. showed that one of the significant independent risk factors for the development of fentanyl- induced cough is young age [10]. However, we have not found any study that has specifically evaluated fentanyl-induced cough in pediatric patients except for two case reports [3,6]. Thus, this study evaluated the effect of IV lidocaine in suppressing fentanyl–induced cough in pediatric patients during induction of general anesthesia.

## Methods

After ethics committee approval and parent/guardian consent, 186 children of either sex, aged between 4 and 10 years, ASA physical status I and II, and scheduled for elective surgery were recruited for the study. The exclusion criteria were body weight exceeding 20% of the ideal body weight, a history of bronchial asthma and chronic obstructive pulmonary disease, respiratory tract infection during the last 4 weeks, children with psychological or emotional disorders and development delay, and with malformation of the tongue and oropharynx. All patients received an oral administration of 0.3 mg/kg of injectable midazolam mixed with a double volume apple juice 30 minutes before separation from parents or IV (1 mg) and atropine (0.02 mg/kg) when IV access was established before the induction of anesthesia. Upon arrival in the operating room (OR), intravenous access was established, standard monitoring including electrocardiography (5 leads), noninvasive blood pressure, pulse oximetry and capnography were connected, and the baseline vital parameters were noted. All patients were preoxygenated with 100% O2 for 5 minutes. Patients were randomly allocated using the sealed envelope technique in three groups of 62 each to receive: 1.0 mg/kg lidocaine (Gr I), 0.5 mg/kg lidocaine (Gr II) or placebo -equal volume of 0.9% saline (Gr III), over 5 s 1 min before the IV administration of 2–3 μg/kg fentanyl during induction of general anesthesia. A blinded observer, who was unaware of the type of medication given to the patients, recorded the number of coughing episodes. Severity of coughing was graded based on the number of episodes of cough (mild, 1–2; moderate, 3–4; and severe, 5 or >5).

Data processing was done with the statistical package R. The statistical parameters index structure, arithmetic mean, standard deviation, minimum and maximum values were calculated. One-Way ANOVA with post-hoc testing was used to test parametric data, whereas χ2-test and Kruskal Wallis test with post-hoc testing were used for nonparametric data. A *P* value of <0.05 was considered statistically significant.

## Results

There was no statistically significant difference between the three groups with regard to age, weight, sex, and ASA class (*P* > 0.05). Table 1. Demographic patient characteristics involved in the research Table 2. Incidence of coughing and its severity in groups. The highest frequency of coughing was found in the placebo group (GR III), with 43.5% having cough, of which 4.8% (3 patients) had severe cough. In Group II, 22.6% had cough of which 1.6% (1 patient) had severe cough, whereas in Group I 16.1% had cough and none had severe cough. With the Chi-square test, we have gained significant difference in the incidence of cough between the first and the third groups (*P* <0.01), and between the second and the third groups (*P* <0.05). According to the severity of coughing between groups we have not gained the statistically significant difference (*P* > 0.05).

**Table 1:** Demographic patient characteristics involved in the research

		**Group I n = 62**	**Group II n = 62**	**Group III n = 62**	
Gender	F	33 (53.2%)	32 (51.6%)	33 (53.2%)	*P* = 0.982
N (%)	M	29 (46.8%)	30 (48.4%)	29 (46.8%)	
Age (year)	Mean ± SD	6.8 ± 2.0	7.0 ± 2.2	7.1 ± 2.2	*P* = 0.692
	Rank	4-10	4-10	4-10	
Weight (kg)	Mean ± SD	24.5 ± 4.6	25.6 ± 5.0	25.7 ± 4.7	*P* = 0.261
	Rank	16 - 35	16 - 36	17 - 36	
ASA	I	52 (83.9%)	49 (79.0%)	53 (85.5%)	*P* = 0.716
N (%)	II	10 (16.1%)	13 (21.0%)	9 (14.5%)	

**Table 2:** Incidence of coughing and its severity in groups

**Groups**		**Patients had no cough**	**Cough severity (%)**			
			**Total**	**Mild**	**Moderate**	**Severe**
Group I	N	52	10	8	2	0
	%	83.9	16.1	12.9	3.2	0.0
Group II	N	48	14	10	3	1
	%	77.4	22.6	16.1	4.8	1.6
Group III	N	35	27	19	5	3
	%	56.5	43.5	30.6	8.1	4.8

Incidence of cough Gr. I vs. Gr. III, *P* = 0.002; Gr. II vs. Gr. III, *P* = 0.022.

Cough severity *P* = 0.441.

## Discussion

In our study, we have shown that administration of 2–3 μg/kg fentanyl through a peripheral venous line induced reflex cough in 43.5% of patients in the placebo group, 22.6% in the 1-mg/kg lidocaine group, and 16.1% in the 0.5-mg/kg lidocaine group. Phua et al. reported that 1.5 μg/kg fentanyl given through a peripheral vein elicited cough in 28% of the patients and a similar incidence of cough was observed by Agarwal et al. following 2 μg/kg IV fentanyl through the same route over a period of 5 seconds [2,4]. Bohrer et al. observed a 45% incidence of cough when 7 μg/kg fentanyl was administered through a central venous catheter over 1 second, whereas a 46% incidence was reported by Lui et al. with 5 μg/kg fentanyl administered through a peripheral vein over 5 seconds [1,11]. In all the above studies, benign cough has been reported, which is consistent with our results. However, Tweed and Dakin reported a case of explosive coughing in a 7-yr-old boy with Trisomy-21 syndrome, after peripheral injection of IV fentanyl (2 μg/kg) that produced periorbital petechiae and was only relieved after induction of anesthesia [3]. Also, Ambesh et al. reported a known case of arteriovenous malformation of tongue and oropharynx of a 12-year-old patient with severe spasmodic cough after receiving IV fentanyl (50 μg), which led to massive engorgement of the tongue and hypopharynx that caused acute airway obstruction and severe hypoxia [6]. In our study, we excluded patients with Trisomy-21 syndrome and with any malformation in the oral cavity or pharynx. However, it was interesting that one patient in Group II and two of three patients in Group III, who were classified as having severe cough, presented with hypertrophied tonsils without local inflammation. There are various hypotheses that try to explain the mechanism of fentanyl-induced cough. According to some studies, fentanyl may inhibit central sympathetic outflow causing vagal predominance, which could trigger cough and reflex bronchoconstriction [4,9,12].

However, the involvement of a vagal-dependent pathway was not supported by some studies because atropine failed to suppress cough [2,9,10]. In our study, we have used atropine before fentanyl in all groups, so we were not able to evaluate the effect of atropine in suppression of fentanyl-induced cough. Additionally, a possible mechanism of fentanyl-induced cough is a pulmonary chemoreflex mediated by either irritant receptors or by vagal C fibre receptors that are close to pulmonary vessels [1,13]. Effective suppression of the cough response from 43% to 3% after terbutaline and salbutamol inhalation supports the concept of bronchoconstriction [11]. Also, suppression of cough with betamethasone inhalation supports the trigger stimulus and bronchial hyperirritability theory [3,4,11]. According to Kamei et al. pretreatment with fentanyl significantly increased the number of citric acid-induced coughs in mice, and this effect was antagonized by pretreatment with moguisteine, a rapidly adapting receptor antagonist, which suggest that fentanyl activates mainly rapidly adapting receptors, but not C-fibers, to enhance citric acid-induced cough [14]. The release of histamine, leukotrienes, interleukins, and other inflammatory mediators from mast cells in the lungs and the possible stimulation of irritant receptors in the tracheobronchial wall are other possible mechanisms of fentanyl-induced cough [4,15,16].

Has been reported that fentanyl did not induce the release of histamine in plasma [17], even during incubation of human skin mast cells with fentanyl [18]. But, Kamei et al. also observed that fentanyl markedly increased the histamine levels in bronchoalveolar lavage fluid (BALF), and this suggest that histamine may enhance cough receptor sensitivity through the activation of histamine H1 receptors in the airways, and it is possible that mast cell heterogenity may play a role in the different effects of fentanyl on histamine release [14].

There are so many clinical studies showed that IV lidocaine before fentanyl administration during induction in general anesthesia, suppress fentanyl-induced cough significantly [5,7,8,19,20]. The precise mechanisms by which intravenous lidocaine prevent fentanyl-induced cough are not clear. There are 4 groups of airway sensory receptors innervated by vagus nerve: slowly adapting receptors (SARs), rapidly adapting receptors (RARs), high-threshold Aδ-receptors (HTARs)and C-fiber receptors (CFRs) [21-24], that participate in various reflexes such as coughing and sneezing, and in respiratory and cardiovascular performance [21,22]. Mechanosensors (SARs and RARs) are suppressed whereas the chemosensors (CFRs and HTARs) are stimulated by lidocaine [25].

It has been proposed that depression of brain stem functions by lidocaine may be responsible for cough suppression or lidocaine may act by anesthetizing peripheral cough receptors in the trachea and hypopharynx [26]. Although the bronchodilating effect of lidocaine has not been confirmed, the intravenous administration of lidocaine suppress mechanically and chemically induced airway reflexes, including the cough reflex [6,8,15,26,27].

The airways are innervated by C-fibers, which express voltage-gated Na^+^ channels with sensitivity or resistance to tetrodotoxin (TTX). Kamei et al. indicate that sodium channels, mainly TTX-resistant sodium channels, may play an important role in the enhancement of C-fiber-mediated cough pathways [28]. However, the role of TTX-resistant sodium channels in the cough reflex is not well understood.

Lidocaine is showed to be effective antitussive agent who blocks sensory neuron voltage-gated sodium channels and suppresses action potential generation and propagation of neurons, the mechanism of action likely involves a reduction in action potential formation evoked by a variety of stimuli in several airway afferent nerve subtypes [29].

Although precise mechanisms of how fentanyl induces cough and lidocaine prevents fentanyl- induced cough are not yet clear, our results demonstrate that IV lidocaine can prevent markedly, fentanyl-induced cough in pediatric patients during induction of general anesthesia even in doses of 0.5 mg/kg (77.4%).

## Competing interests

The authors have nothing to disclose and they have no *f*inancial nor non-financial competing interests.

## Authors’contributions

ZN participated in the design of the study and language correction. VJ participated in its design and coordination and helped to draft the manuscript. MG performed the statistical analysis. I guarantee that the manuscript has not been published before and has not been submitted for publication elsewhere. The manuscript does not contain any unlawful statements and it does not violate any rights of others. All authors read and approved the final manuscript
